# Climate Change Synchronizes Growth and iWUE Across Species in a Temperate-Submediterranean Mixed Oak Forest

**DOI:** 10.3389/fpls.2020.00706

**Published:** 2020-06-11

**Authors:** Isabel Dorado-Liñán, María Valbuena-Carabaña, Isabel Cañellas, Luis Gil, Guillermo Gea-Izquierdo

**Affiliations:** ^1^Forest Research Centre, Instituto Nacional de Investigación y Tecnología Agraria y Alimentaria (INIA-CIFOR), Madrid, Spain; ^2^Forest Genetics and Ecophysiology Research Group, E.T.S. Forestry Engineering, Universidad Politécnica de Madrid, Madrid, Spain

**Keywords:** hybrid oak, water-use efficiency, tree rings, *Quercus petraea*, *Quercus pyrenaica*, tree growth, interspecific hybridization, climate change

## Abstract

Tree species have good tolerance to a range of environmental conditions, though their ability to respond and persist to environmental changes is dramatically reduced at the rear-edge distribution limits. At those edges, gene flow conferring adaptation is impaired due to lack of populations at lower latitudes. Thus, trees mainly rely on phenotypic changes to buffer against long-term environmental changes. Interspecific hybridization may offer an alternative mechanism in the generation of novel genetic recombinants that could be particularly valuable to ensure persistence in geographically isolated forests. In this paper, we take advantage of the longevity of a temperate-submediterranean mixed-oak forest to explore the long-term impact of environmental changes on two different oak species and their hybrid. Individual trees were genetically characterized and classified into three groups: pure *Quercus petraea* (Matt.), Liebl, pure *Q. pyrenaica* Willd, and hybrids. We calculated basal area increment and intrinsic water-use efficiency (iWUE) from tree-ring width and δ^13^C per genetic group, respectively. Tree-growth drivers were assessed using correlation analyses and generalized linear mixed models for two contrasting climatic periods: (1880–1915, colder with [CO_2_] < 303 ppm; and 1980–2015, warmer with [CO_2_] > 338 ppm). The three genetic groups have increased radial growth and iWUE during the last decades, being the least drought-tolerant QuPe the most sensitive species to water stress. However, no significant differences were found among genetic groups neither in mean growth rate nor in mean iWUE. Furthermore, little differences were found in the response to climate among groups. Genetic groups only differed in the relationship between δ^13^C and temperature and precipitation during the earlier period, but such a difference disappeared during the recent decades. Climate change may have promoted species-level convergence as a response to environment-induced growth limitations, which translated in synchronized growth and response to climate as well as a tighter stomatal control and increased iWUE across coexisting oak species.

## Introduction

Forest persistence largely depends on the ability of individual species to adapt their hydraulic strategy to regimen shifts by changes in genotype and phenotype (Potvin and Tousignant, 1996; Aitken et al., 2008; Kremer et al., 2012). Genotypic changes take place through gene frequency changes across generations and are slower than phenotypic changes (Alberto et al., 2013). Although local adaptation (Kawecki and Ebert, 2004) is more common in trees than in some other non-woody plant species, the long generation and residence times in the same environment challenges tree survival under rapid and abrupt changes in climate (Savolainen et al., 2007). However, the long life-span of trees provides a wealth of information on the evolutionary phenotypic adjustments to environmental changes using subrogates from which past adaptation of trees to climate changes can be inferred (e.g., Fritts, 1976).

Trees generally express good tolerance to a range of environmental conditions and large plastic responses (e.g., Sáenz-Romero et al., 2017). The capacity of forest species to respond and persist to environmental change is conferred by three mechanisms: adaptation, migration and phenotypic plasticity (Jump and Peñuelas, 2005; Aitken et al., 2008). In the northern hemisphere, at the retreating edge of the species distributions (also named rear-edge), adaptation through gene flow is limited since there are no populations further south. Thus, at those sites, species persistence may be seriously compromised if climate change rate is beyond migration and/or phenotypic adaptability rates (Gomulkiewicz and Holt, 1995; Hampe and Petit, 2005; Hampe and Jump, 2011). The question is also whether phenotypic changes are able to buffer plants against long-term environmental changes on forests at the tolerance limits, considering that genotypic changes do not have time to take place unless rear-edge populations hybridize with other species. In this context, interspecific hybridization could be particularly valuable in long-lived forests and populations geographically isolated due to the limited accumulation of novel genetic variations for an adaptative response as a consequence of generation length, reduced mutation rates and limited migration across heterogeneous environments (Davis and Shaw, 2001; Petit and Hampe, 2006; Kremer et al., 2012). Thus, interspecific hybridization has been recognized as a relevant mechanism in the generation of novel genetic forms that may ensure populations persistence in a changing climate (Anderson and Stebbins, 1954; Rius and Darling, 2014; Janes and Hamilton, 2017).

The central Iberian Peninsula conforms the equatorial edge of distribution of temperate deciduous species such as *Quercus petraea* (Mattuschka) Liebl., which coexists with more drought tolerant submediterranean deciduous oak species such as *Quercus pyrenaica* Willd. Oaks (genus *Quercus*) are among the plant species showing natural hybridization across species (Rushton, 1993) with different levels of gene flow among species (Curtu et al., 2007; Valbuena-Carabaña et al., 2007). Hybridization between *Q. petraea* and *Q. pyrenaica* may confer the hybrids with a full array of advantageous adaptation traits which may explain the recently reported lack of differences in growth rates between temperate and submediterranean oaks in mixed old growth forest at the temperate rear-edge (i.e., Dorado-Liñán et al., 2017). However, whether the lack of difference in growth rates is the result of inter-specific hybridization or phenotypical adaption is rather unclear since most of the studies comparing performance among individuals genotypically characterized have been carried using young individuals growing in experimental fields (e.g., Ponton et al., 2002; Brendel et al., 2008; Roussel et al., 2009a, b; Abadie et al., 2012).

Tree growth is a sensitive and useful indicator of species vitality and fitness (Fritts, 1976; Dobbertin, 2005) and may allow inferences on past multispecies forest dynamics and changes in species dominance (e.g., Dorado-Liñán et al., 2017). The analysis of growth chronologies derived from natural old forests allows reconstructing forest and individual species history in response to long-term environmental changes, as well as inferring the degree of adaptability of the different species. In addition, stable carbon isotopes in tree-rings are effective proxies to track ecophysiological changes in plant carbon and water relations at large spatiotemporal scales (Saurer et al., 2014; Frank et al., 2015; Gea-Izquierdo et al., 2017; Martínez-Sancho et al., 2018). Indeed, the interannual variation in δ^13^C have been related to leaf-level physiological processes (e.g., Andreu-Hayles et al., 2011; Shestakova et al., 2014; Martínez-Sancho et al., 2018), which is particularly relevant at the retreating edge where trees are thriving under sub-optimal conditions and growth patterns may not be as informative as in the range core (Cavin and Jump, 2016; Hacket-Pain et al., 2016). This information is particularly well suited not only to provide evidence on how forest trees are likely to respond to forthcoming climate changes, but also to increasing atmospheric CO_2_ that may increase tree growth due to the so-called “fertilization effect” (McDowell et al., 2010; Saurer et al., 2014; Martínez-Sancho et al., 2018). Such dual approach has revealed both coherent and diverging responses to environmental changes depending on the site and species (Andreu-Hayles et al., 2011; Martínez-Sancho et al., 2018) but has not been used to infer levels of adaptability and physiological response to environmental variability of pure species versus hybrids.

In this study, we characterized the functional drivers (as inferred through δ^13^C and tree-ring growth) of two co-occurring temperate and submediterranean oak species as well as their genetic hybrid in a mountain forest in the central Iberian Peninsula during two periods of contrasting climate and atmospheric CO_2_ concentration. To this end, three chronologies of centennial trees spanning the 19th and 20th centuries comprising *Q. petraea*, *Q. pyrenaica*, and the genetic hybrid of these two species were used. We expect that the response of the pure species to climate will be structured following a physiological gradient from the more tolerant to drought (i.e., *Q. pyrenaica*) to the less tolerant to drought (i.e., *Q. petraea*) species and that differences among species have increased during the last century as a consequence of climate change. The species-related response to climate will be assessed based on (i) establishing the climate–growth relationship that will theoretically show how species with lower drought-tolerance display a greater responses to moisture availability; (ii) assessing the climate drivers of the ^13^C discrimination and rate of intrinsic water-use efficiency (iWUE), that will presumably be related to the level of conservatism in the species’ hydraulic strategy; and (iii) testing the potential climate-induced increased or decreased inter-species synchrony in growth by modeling basal area increment (BAI) as a function of climate and iWUE in two contrasting periods: 1880–1915 and 1980–2015. To our knowledge, this is the first attempt of characterizing differences in growth and iWUE among individual trees that have been genetically characterized (and therefore unequivocally assigned to a pure species or hybrid forms) in a natural temperature-submediterranean mixed oak forest.

## Materials and Methods

### Site Description

**FIGURE 1:**
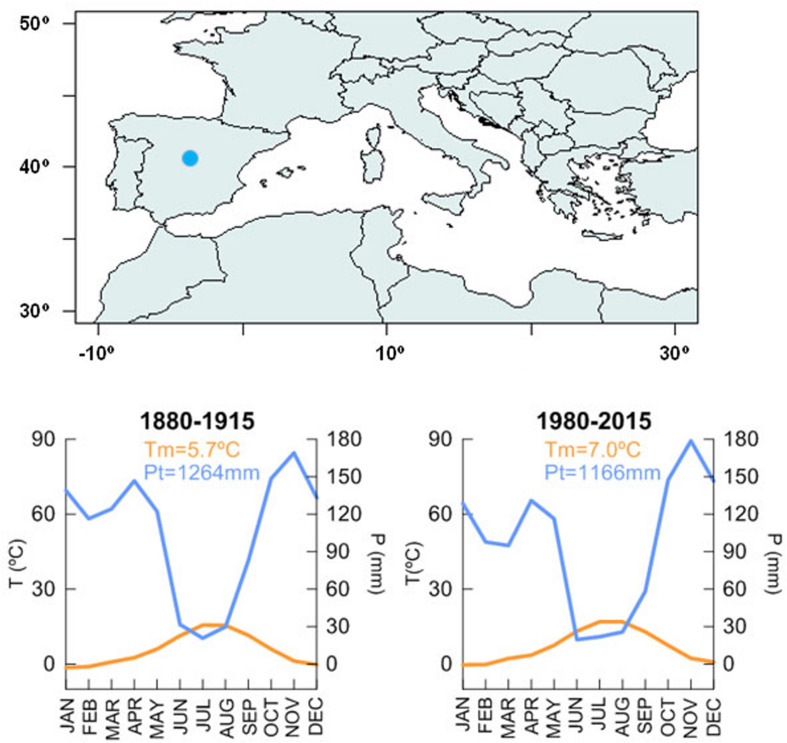
Location of Somosierra mixed-oak forest. Mean monthly temperature and monthly sum precipitation for the two periods under study: 1880–1915 and 1980–2015. Long climate data is derived from the Brunet et al. (2007) and scaled to Navacerrada meteorological station.

The study site, Somosierra, is a mixed-oak forest located around 1,500 m a.s.l. in the center of the Iberian Peninsula, in the so-called Central System Range (Figure 1). The climate is Mediterranean continental (Oromediterranean humid according to Rivas-Martínez, 1983) with a current mean annual temperature of 7.0°C and the annual sum of precipitation over 1,100 mm for the period 1980–2015 with a summer dry period spanning June, July, and August. Climate at Somosierra was slightly colder and wetter at the end of the 19th century and beginning of the 20th century, when the mean annual temperature was 5.7°C and total annual precipitation above 1,200 (period 1880–1915). This means that in the last 100 years, mean annual temperature has increased 1.3°C and annual sum of precipitation has decreased almost 100 mm.

The study site is an open oak forest (227 trees/ha, 18.5 m^2^ ha^–1^) dominated by *Quercus petraea* (Matt.), Liebl. and *Quercus pyrenaica* Willd (QuPe and QuPy hereafter) and a wide range of morphological intermediate individuals with different levels of hybridization (Valbuena-Carabaña et al., 2005, 2007). The hybrid *Quercus petraea* × *Quercus pyrenaica* is named *Quercus* × *trabutii* (Hy, 1895) or *Quercus* × *legionensis* (Vicioso, 1950) and will be referred as QuHy hereafter. In addition, there is presence of other species such as *Ilex aquifolium* L., *Prunus avium* L., *Betula pendula* Roth, *Corylus avellana* L., *Sorbus aria* (L.) Crantz and *Sorbus aucuparia* L. This low-density forest dominated by oak species has been traditionally managed to produce pasture and forage for livestock and the result is the current open canopy forest structure.

QuPe is a typical temperate species reaching at this latitude its southernmost distribution limit. Conversely, QuPy is a submediterranean oak species growing at the geographical core of the species distribution range. QuPy has shown a larger osmoregulation capacity and higher ability to cope with water stress than QuPe (Aranda et al., 2004) and hence, QuPy is described as better adapted to submediterranean climate than temperate oaks. Yet, QuPe is considered more-drought tolerant than other co-occurring temperate species such as European beech (*Fagus sylvatica* L.; Raftoyannis and Radoglou, 2002; Aranda et al., 2005; Lendzion and Leuschner, 2008). Despite the theoretical physiological classification, recent studies described a similar performance of QuPe and submediterranean oaks (QuPy) coexisting in an oak-beech forest located at the temperate species’ rear-edge nearby the study area (Dorado-Liñán et al., 2017).

Trees in a 20-ha plot in Somosierra were characterized and classified in phenotypic and genetic groups in Valbuena-Carabaña et al. (2007). QuPe usually displays glabrous to slightly hairy round-shaped leaves, whereas QuPy presents densely hairy and lobulated leaves with deep sinuses and big lobes. In Valbuena-Carabaña et al. (2007), principal component analysis was used to summarize 15 leaf-morphology traits, a discriminant linear function was constructed, and posterior probability was used to classify individuals into pure types or putative hybrids showing intermediate leaf-morphology. The assignation of trees to pure types or hybrids was performed considering a probability level of 0.85. High proportion of individuals displayed intermediate phenotypes, so in order to assess genetic admixture between both species, all individuals underwent a genetic characterization using nine nuclear microsatellites (QpZAG9, QpZAG36, QpZAG110, QrZAG5, QrZAG7, QrZAG11, QrZAG39, MSQ4, and MSQ13) and molecular marker scores were used to distinguish between pure and hybrids. Individuals were probabilistically assigned to species by means of a Bayesian model-based clustering method described in detail in Valbuena-Carabaña et al. (2005, 2007). For this study, we established three genetic groups: pure QuPe individuals, pure QuPy individuals and hybrids displaying between 15 and 85% of admixture (QuHy). Those trees with the most congruent classification at both morphological and genetic levels were selected, although hybrids formed the genetic group showing lower agreement between the genetic and phenotypic classifications (see Table 1).

**TABLE 1:** Proportion of phenotypes (columns) in each defined genetic group (rows): pure *Q. petraea* (QuPe), pure *Q. pyrenaica* (QuPy), and the hybrids between the two of them (QuHy).

**Genotype/Phenotype**	**Ph_QuPe**	**Ph_QuHy**	**Ph_QuPy**
QuPe	82%	12%	6%
QuHy	22%	33%	45%
QuPy	0%	0%	100%

### Sampling and Chronologies Development

During winter of 2014 and spring and summer of 2015, 20 dominant or co-dominant trees including different size classes were sampled per each genetic group (i.e., a total of 60 trees). Diameter at breast height (DBH) and total height were measured in every tree and two-to-three cores of 5 mm of diameter were taken. Cores were air-dried and sanded until ring boundaries were clearly visible under a stereo-microscope and samples were visually crossdated following Stokes and Smiley (1968). The ring-width of each sample was measured with a precision of 0.001 mm using a Linntab 6 (RINNTECH) measuring device. The correct dating of every sample was re-checked using COFECHA (Holmes, 1983). Individual tree-ring series were converted to BAIs to account for age-related growth trends in non-juvenile tree ring-width series (Biondi and Qaedan, 2008). The conversion of the individual ring-width measurements to individual-BAI series follows the equation:

BAI=tπ(r-t2r)t-12

where *r* is the tree radius and *t* is the year of tree-ring formation.

The final mean chronologies were built by averaging individual BAI series. The chronologies statistical quality was checked using the expressed population signal (EPS; Wigley et al., 1984) and a threshold value of EPS > 0.85 was considered reliable. The common period of all three BAI chronologies displayed an EPS > 0.85.

### Development of δ^13^C Chronologies

For isotope measurements, five representative and undamaged cores were selected per genetic group (i.e., a total of 15 trees). Tree-rings were split using a scalpel under a stereomicroscope for two equally long 30-year periods and avoiding juvenile years: 1880–1915 and 1980–2015. These two periods were selected to compare tree growth and iWUE previous to current global warming and under lower atmospheric CO_2_ concentration (1880–1915) and growth and iWUE during the warmest period in the last 150 years (Brunet et al., 2007) and under current CO_2_ enriched atmosphere. Specifically, the atmospheric CO_2_ concentration has increased from a range of 290–302 ppm during 1880–1915 to 338–400 ppm during 1980–2015 and is considered one of the main contributors to the recent global warming (IPCC, 2014). The individual tree rings were split and then homogenized using a milling device and an accurate weight of wood powder was loaded in a tin capsule. Measurements of stable carbon isotopes were undertaken by combustion using an elemental analyzer coupled online via open split in the Stable Isotope Facility UC Davis. The isotope ratios are given in the delta (δ) notation, relative to the standard VPDB (Vienna-PDB).

The isotope discrimination (Δ) between plant material (δ^13^Cplant) and atmospheric CO_2_ (δ^13^Catm) is defined as Δ = (δ^13^Catm − δ^13^Cplant)/(1 + δ^13^Cplant/1,000), which corrects for the increasing concentration in atmospheric CO_2_ due to fossil fuel burning (i.e., *Suess* effect, Tans et al., 1979). According to Farquhar et al. (1982), Δ in C_3_ plants can be related to plant physiological properties approximately via the linear relationship Δ = *a* + (*b* − *a*) × *Ci*/*Ca*, where *a* is the fractionation associated with the diffusion of CO_2_ through the stomata (*a* ≈ 4.4‰; O’Leary, 1981), *b* is the constant associated with fractionation during carboxylation (*b* ≈ 27‰; Farquhar and Richards, 1984), and *Ci* and *Ca* are the intercellular and atmospheric concentrations of CO_2_ (μmol mol^–1^), respectively. For the calculation of Δ for tree-rings, δ^13^C-values of the atmosphere (δ^13^Catm) need to be corrected over the last two centuries due to the combustion of fossil fuels. These values were obtained combining data from Mauna Loa records^1^ and McCarroll and Loader (2004). In addition, we calculated annual estimates of iWUE per genetic group using the δ^13^C series. iWUE approximates the ratio of the assimilation rate (*A*) and stomatal conductance of water vapor (*g*_*H*__2__0_):

$$i\,W\,U\,E=\frac{A}{g\,H_{2}\,O}≅\frac{g\,C\,O_{2}\,\left(C\,a-C\,i\right)}{g\,H_{2}\,O}≅\frac{1}{1.6}\,\left(C\,a-C\,i\right)$$

Individual δ^13^C series were corrected for the increasingly δ^13^C depleted atmospheric CO_2_ due to fossil fuel burning since the beginning of industrialization (Tans et al., 1979). Annually resolved chronologies of δ^13^C and iWUE were obtained for each of the three genetic groups and for the two common periods 1880–1915 and 1980–2015 by averaging individual series.

### Climate Data, Statistical Analyses, and Model Fitting

Time series of BAI and iWUE were investigated to assess the short- and long-term (i.e., inter annual to multi decadal) changes in growth among genetic groups. Linear trends were calculated for both subperiods under study (1880–1915 and 1980–2015) using least-squares regressions. Meteorological homogenized records of mean monthly temperatures and precipitation spanning the period 1946–2014 were available from a nearby station (Navacerrada) located at 1,894 m a.s.l. Additional long meteorological temperature and precipitation records spanning the period 1860–2005 were available from Madrid (Brunet et al., 2006). Both records, Navacerrada and Madrid displayed almost identical interannual and decadal variations, but they differ in the absolute values (not shown). Thus, in order to obtain a long record for Navacerrada, the Madrid record was corrected for the differences in absolute values and rescaled to the mean of the high-mountain record. The long chronologies and meteorological records allowed to study the influence of climate on tree growth and δ^13^C in two periods separated 100 years: 1880–1915 and 1980–2015. Such analysis allowed for the characterization of differences among genetic groups as well as the evolution of those differences during the last century.

The influence of monthly and seasonal temperature and precipitation on BAI and δ^13^C was studied by means of Pearson’s correlation coefficients. The differences among genetic groups in BAI and iWUE were evaluated using the Kruskal–Wallis test and the Nemenyi-test for the pairwise comparisons. A non-parametric test was chosen since the data was not normally distributed in most of cases.

Generalized linear mixed-effects models (GLMM) were fitted to explain BAI as a function of iWUE and climate for the two periods under study: 1880–1915 and 1980–2015. The GLMM approach was chosen since the BAI dataset better approximated a Gamma distribution (Gea-Izquierdo and Cañellas, 2009; Dorado-Liñán et al., 2019). One global GLMM was developed including all trees and genetic groups and considering different combinations of fixed and random factors. As fixed effects we considered iWUE, seasonal climate variables and individual tree features such as current DBH and tree height (see Supplementary Table S1). In order to test whether the influence of iWUE and climatic variables on growth is different depending on individual tree, we tested random intercepts aiming at characterizing idiosyncratic variations related to individual tree response to iWUE and seasonal climate variables. Starting from a saturated GLMM model, we created the fully crossed set of models including different random and fixed effects and selected the best and most parsimonious model. Model selection and reduction of explanatory variables was done based on the Akaike’s information criteria (Akaike, 1974) corrected for small sample size (AIC) with lower AIC values indicating a model’s higher explanatory power. The significant contribution of the random effects included in the selected GLMM was confirmed using Likelihood Ratio Tests. Absence of collinearity among predictors was checked by the variance inflation factor (VIF; Zuur et al., 2009) and variables displaying a VIF over two were discarded from the final model. All analyses were done using R software version 3.2.3 (R Core Development Team, 2015) and packages dplR (Bunn, 2008), forecast (Hyndman and Khandakar, 2008), PMCMR (Pohlert, 2014), lme4 (Bates and Maechler, 2009), MASS (Venables and Ripley, 2002), MuMIn (Bartoń, 2011), and mlmRev (Bates et al., 2014).

## Results

### Species Co-existence and Growth

Most trees genetically classified as QuPy and QuPe also displayed a phenotype consistent with the species, whereas trees belonging to the hybrids QuHy displayed similar proportions of phenotypes of pure species and hybrids (Table 1).

**FIGURE 2:**
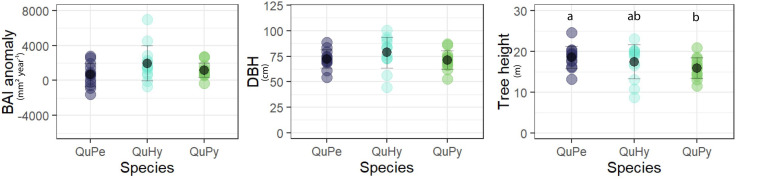
Comparison of different attributes among genetic groups (i.e., the two pure species and the hybrid). BAI anomaly (mm^2^ year^– 1^): difference between mean growth during the period 1980–2015 respect to that of the period 1880–1915; DBH (cm): mean diameter at breast height. Different letters indicate significant differences at 0.05 level.

Comparing size and growth rate among the three genetic groups revealed no statistical differences on BAI anomaly and DBH (Figure 2). However, QuPe trees were statistically taller (*p* < 0.05) than those of QuPy, laying the height of QuHy in between the two pure species.

**FIGURE 3:**
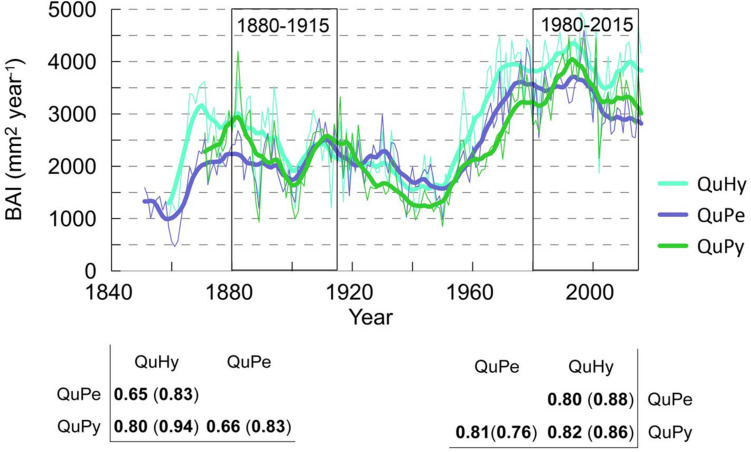
A Basal area increment chronologies (BAI) of *Quercus petraea* (QuPe), *Quercus pyrenaica* (QuPy) and hybrid *Quercus* (QuHy). Bottom panel displays the correlation among BAI series for the two subperiods. Values in brackets correspond to the value of correlation for 10 years high-pass filtered series with a centered moving average. Bold font denotes significant correlation at 99% level.

**FIGURE 4:**
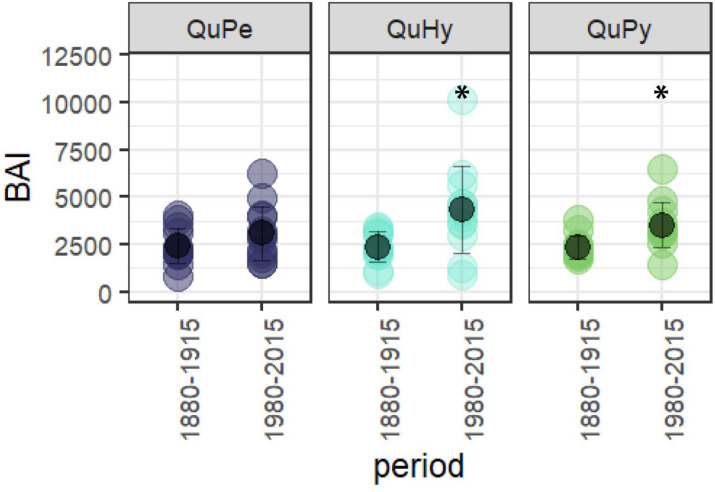
Differences in BAI between the first and the second subperiod for the three genetic groups. Asterisk (^∗^) denotes significant differences among groups at 0.99 significance level.

The total length as well as the reliable period of the three BAI chronologies were similar, being the QuPe chronology the longest (Table 2 and Figure 3). The reliable period covered by all three chronologies spanned from 1866 to 2015 and the comparison of growth curves revealed a synchronized growth pattern among species. QuHy displayed the highest growth rates at the two subperiods: 1880–1915 and 1980–2015 (2,326 and 3,920 mm^2^ year^–1^, respectively) (Table 2), whereas QuPe displayed the lowest mean BAI for both subperiods (2,093 and 3,221 mm^2^ year^–1^, respectively). However, these differences in growth rates among species were non-significant (Table 2 and Supplementary Figure S1). All three genetic groups displayed lower growth rates than average during the period 1880–1915 (Table 2 and Figure 3). In contrast, growth rates during the period 1980–2015 were higher than those of 1880–1915, although the increase in growth was only significant for QuPy and QuHy (Figure 4). The higher mean growth was coupled to significant decreasing growth trend in pure species (i.e., QuPe and QuPy) and such growth decline came along with a reduction in the inter-tree correlation within each genetic group (Table 2): all three groups showed a higher mean correlation among trees during the first period than during the second one. Despite this, the correlation among the three BAI chronologies increased during the second period respect to the first one (Figure 3).

**TABLE 2:** Characteristics of BAI chronologies for the two analyzed periods 1880–1915 and 1980–2015.

**Genetic group**	**Trees/cores**	**Total time span**	**Reliable time span**	**1880–1915**			**1980–2015**		
				***r***	**Mean ± SD**	**Slope**	***r***	**Mean ± SD**	**Slope**
QuPe	18/34	1817–2015	1846–2015	0.28	2,039 ± 439	12.1	0.23	3,221 ± 535	**−30.0**
QuHy	19/35	1826–2015	1854–2015	0.40	2,326 ± 635	−13.6	0.29	3,920 ± 612	−16.3
QuPy	20/39	1830–2015	1866–2015	0.35	2,179 ± 606	12.9	0.31	3,446 ± 568	**−25.5**

**r*, mean correlation among individual series. Reliable time-span refers to portion of chronology with EPS > 0.85. The rates of change per year are calculated as the slopes of the linear regressions. Significant slopes (*p* < 0.01) are in bold.*

### Changes in δ^13^C and iWUE

**FIGURE 5:**
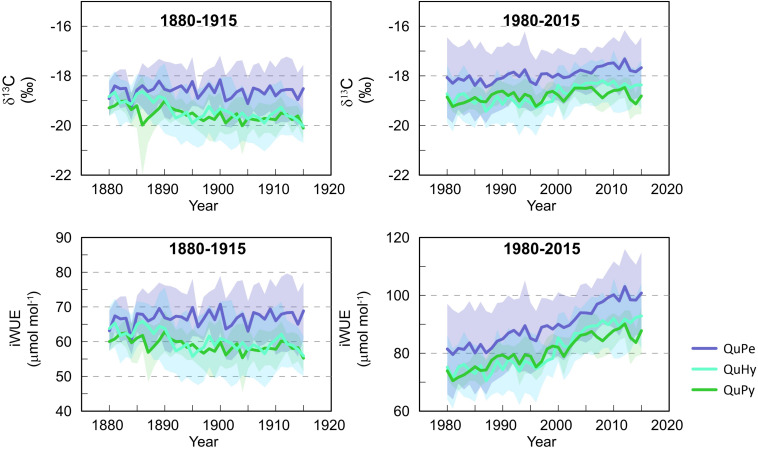
Variations in δ^13^C (**upper panels**) and iWUE (**lower panels**) per genetic group for the two studied periods 1980–1915 (**left panels**) and 1980–2015 (**right panels**). Lines represent the arithmetic mean of the individual tree values per genetic group. Shaded area represents one standard deviation from the mean.

The temperate oak QuPe displayed higher mean δ^13^C and iWUE compared to QuHy and QuPy for both subperiods 1880–1915 and 1980–2015 (Table 3 and Figure 5), although differences were statistically non-significant (Supplementary Figure S2). QuHy and QuPy displayed similar mean δ^13^C and iWUE values and significant decreasing and increasing trends for the first and second subperiods, respectively (Table 3 and Figure 5). In contrast, QuPe did not show significant trends in δ^13^C and iWUE during the period 1880–1915 but displayed similar significant increasing growth trends for both variables during the second subperiod analyzed (1980–2015).

**TABLE 3:** Characteristics of the δ^13^C and iWUE chronologies for the two analyzed periods 1880–1915 and 1980–2015.

		**δ^13^C**						**iWUE**					
**Genetic group**	**N trees**	**1880–1915**			**1980–2015**			**1880–1915**			**1980–2015**		
		***r***	**Mean ± SD**	**Slope**	***r***	**Mean ± SD**	**Slope**	***r***	**Mean ± SD**	**Slope**	***r***	**Mean ± SD**	**Slope**
QuPe	5	0.08	−18.6 ± 0.3	0.00	0.18	−18.0 ± 0.3	**0.02**	**0.10**	64.8 ± 2.1	0.04	0.64	81.1 ± 6.8	**0.62**
QuHy	5	0.39	−19.4 ± 0.4	−**0.03**	0.35	−18.8 ± 0.4	**0.03**	0.27	58.1 ± 2.8	−**0.18**	0.73	78.6 ± 7.3	**0.64**
QuPy	5	0.23	−19.6 ± 0.3	−**0.02**	0.12	−18.8 ± 0.2	**0.12**	**0.12**	56.7 ± 2.0	−**0.11**	0.52	77.9 ± 5.3	**0.47**

**r*, mean correlation among individual series; Mean ± SD, mean ± standard deviation; slope, rates of change per year calculated as the slope of the linear regression. Significant slopes (*p* < 0.01) are in bold font.*

**FIGURE 6:**
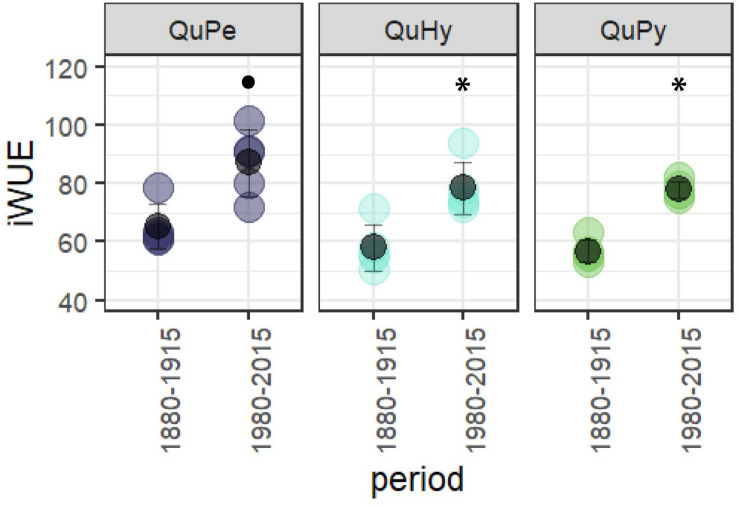
Differences in iWUE between the first and the second subperiod for the three genetic groups. Black filled dot and asterisk (^∗^) denotes significant differences at 0.95 and 0.99 significance level, respectively.

Intrinsic water-use efficiency significantly increased in all three species during the period 1980–2015 respect to the period 1880–1915 (Figure 6), being QuPe the species displaying the highest mean iWUE in both periods.

We observed higher intra-specific (Table 3) and inter-specific (Table 4 and Figure 5) correlations among δ^13^C and iWUE series during the second period than during the first period. This apparent increase in synchronicity within and across genetic groups seems to be largely due to the pronounced significant positive trend in the studied functional traits. This is particularly noticeable in the case of iWUE during the second period in which the mean interseries correlation values per genetic group are much higher than for those of δ^13^C (i.e., series with less pronounced trend) (Table 3). The significant correlation values among chronologies disappeared in most of the cases when high-pass filtering the series (i.e., trend is removed; Table 4) and only δ^13^C chronologies of QuPe and QuHy displayed a significant correlation during the second period.

**TABLE 4:** Correlation among δ^13^C series for the two subperiods.

	**1880–1915**		**1980–2015**		
	**QuHy**	**QuPe**	**QuPe**	**QuHy**	
QuPe	0.17 (0.14)			**0.77 (0.50)**	QuPe
QuPy	**0.50** (0.03)	0.15 (0.11)	**0.53** (0.34)	**0.47** (0.24)	QuPy

*Values in brackets correspond to the value of correlation after removing the trend using a linear regression model. Bold font denotes significant correlation at 99% level. See Supplementary Table S2 for iWUE values.*

### Growth–Climate Relationships

**FIGURE 7:**
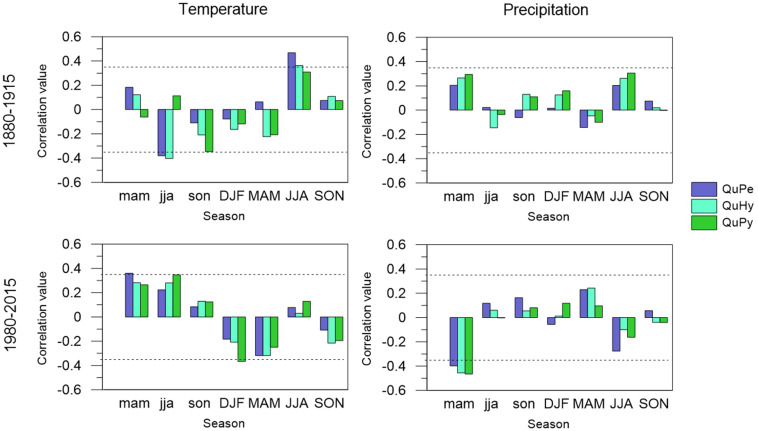
Climate–BAI relationships of QuPe, QuHy, and QuPy with seasonal mean temperature (**left panels**) and precipitation sum (**right panels**) for the two subperiods under study: 1880–1915 (**upper panels**) and 1980–2015 (**lower panels**). Seasonal variables include winter (DJF); spring (MAM); summer (JJA), and autumn (SON) for the previous and current year of growth (low-case and upper-case, respectively). BAI series were smoothed with a 10 years spline. Dashes lines represent 95% significance level.

Tree growth of the three genetic groups was mainly constrained by summer temperature at the end of the 19th century and beginning of the 20th century (Figure 7). Tree growth displayed a significant positive correlation with summer temperature, regardless of the tree species. This effect was more pronounced in the temperate and least drought tolerant QuPe than in QuHy and QuPy. Similarly, previous year summer temperature was negatively related to tree growth in QuPe and QuHy but no relationship was found for QuPy. Precipitation played a minor role and no significant seasonal correlation with tree growth was observed for the period 1880–2015. Conversely, during the most recent decades (i.e., period 1980–2015) the influence of climate decreased compared to the earlier period. The positive relationship between current year summer temperature on tree growth was no longer observed in any of the genetic groups. In turn, previous year spring temperatures became a relevant driver for QuPe growth. Similarly, previous year summer and winter temperatures were positively and negatively correlated to QuPy tree growth, respectively. Previous year spring precipitation was negatively related to growth in all genetic groups.

### δ^13^C-Climate Relationships

**FIGURE 8:**
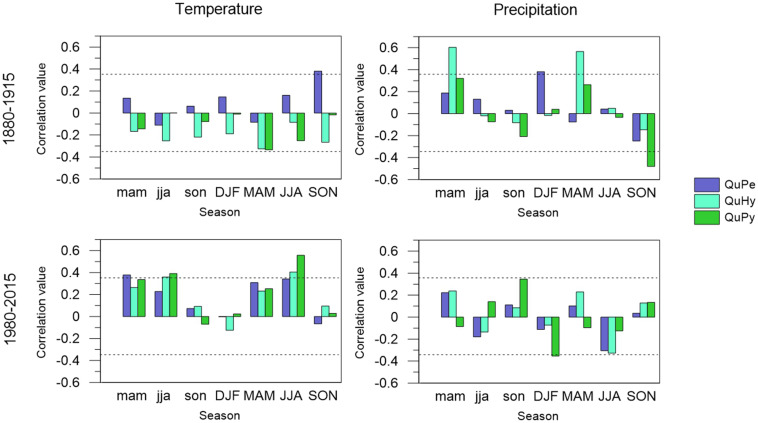
Climate-tree-ring δ^13^C relationships of QuPe, QuHy, and QuPy with seasonal mean temperature (**left panels**) and precipitation sum (**right panels**) for the two subperiods under study: 1880–1915 (**upper panels**) and 1980–2015 (**lower panels**). Seasonal variables include winter (DJF); spring (MAM); summer (JJA), and autumn (SON) for the previous and current year of growth (low-case and upper-case, respectively). Dashes lines represent 95% significance level.

The correlation pattern of δ^13^C variations with temperature and precipitation revealed an increased influence of temperature and a decreased influence of precipitation during the period 1980–2015 respect to the period 1880–1915 (Figure 8). As for growth, the overall influence of temperatures was greater than that of precipitation, with the exception of QuHy for the early period. QuPe was the only species displaying a significant correlation with seasonal temperature during the early period 1880–1915. Concretely, autumn temperatures displayed a significant positive correlation with δ^13^C variations. All three genetic groups displayed significant correlations with seasonal precipitation during the earlier period, but the relationship differed across genetic groups: current year winter precipitation was positively related to δ^13^C of QuPe whereas δ^13^C of QuHy was positively correlated to current and previous year spring precipitation. Conversely, autumn precipitation showed a negative influence on δ^13^C of QuPy.

During the latter period 1980–2015 temperature and particularly summer temperature was the main driver of δ^13^C in all three genetic groups. QuPy, QuHy, and QuPe displayed significant positive correlations with current year summer temperature. QuPy and QuHy also displayed significant positive correlations with previous year summer temperature, whereas for QuPe it was previous year spring temperature the season showing a significant positive effect. The influence of precipitation on δ^13^C variations was very low and only QuPy displayed a positive (negative) influence of (previous year autumn) winter precipitation on δ^13^C.

### Tree Growth Explained by iWUE and Climate

**FIGURE 9:**
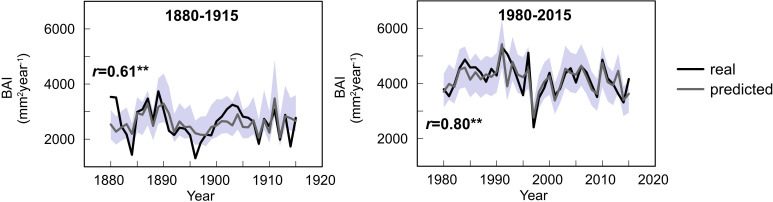
Observed (black) and predicted (gray line) mean chronology of basal area increments for the study sites based on the GLMM developed for each period. Gray area indicates the 95% confidence intervals of prediction. Value of correlation between observed and predicted data is shown. Double asterisk (**) denotes significance at 99% level.

The GLMMs displayed a larger percentage of variance explained for the second period (73%) than for the first period (58%). The predictive skills of both models, understood as the adjusted *R*^2^ between real and simulated data, were good: 0.49 and 0.71 for the first and second periods, respectively, denoting a better performance of the model for the second period. Similarly, the correlation between the real and simulated mean BAI chronology for all three species was 0.61 and 0.80 for the first and second period, respectively (Figure 9), although the predictive skills of the model varied across species (Supplementary Figure S3).

According to the models, tree growth during the period 1880–1915 was controlled by the positive effect of iWUE and current year summer precipitation; and by the negative effect of current year spring precipitation and previous winter and summer temperature (Table 5 and Supplementary Table S3). During the latter period, iWUE was no longer a relevant predictor in the model. In turn, the size of the tree (as estimated by DBH) became a relevant positive predictor of tree growth along with previous year summer climate. In contrast, current year spring temperature and previous year spring precipitation and winter climate negatively affected BAI. Generally, more lag-effects (i.e., legancies/relationships with previous year climatic covariates) were found during the second period than during the first one. The comparison across different GLMM with different random effects revealed that the model accounting for the variation in individual tree response (i.e., including tree ID as a random effect; Supplementary Table S1) to the slope of iWUE, displayed the lowest AIC value. Similar models but including random effects accounting for the variation of climate variables linked to the individual tree, displayed higher AIC values (Supplementary Tables S4, S5).

**TABLE 5:** Fixed effects included in the GLMM with BAI as dependent variable for the first and second period of study, respectively.

**Variable**	**1880–1915**				**1980–2015**			
	**Estimate**	**Error**	***t*-value**	***p*-value**	**Estimate**	**Error**	***t*-value**	***p*-value**
iWUE	0.030	0.008	3.8970	<0.001				
DBH					0.022	0.006	3.441	<0.001
SuP	0.116	0.033	3.5350	<0.001				
SpT					–0.036	0.006	–5.791	<0.001
SpP	–0.046	0.013	–3.6370	<0.001				
WiT	–0.114	0.018	–6.4580	<0.001	–0.046	0.009	–5.244	<0.001
WiP					–0.018	0.004	–4.775	<0.001
SuTt	–0.059	0.015	–3.9340	<0.001	0.039	0.010	4.103	<0.001
SuPt					0.096	0.022	4.463	<0.001
SpPt					–0.040	0.007	–6.045	<0.001
WiTt					–0.016	0.003	–4.770	<0.001

*The table shows the best-guess average effects in the population (Estimate) along with corresponding error, *t*-value and *p*-values for each subperiod. Redundancy of covariates was checked by excluding those with VIF > 2. Abbreviations as in Supplementary Table S1.*

## Discussion

### No Statistical Evidence of Species-Specific Differences in Growth and iWUE

The studied mixed-oak forest is characterized by high hybridization rates between submediterranean and temperate oak species compared to similar nearby populations (Valbuena-Carabaña et al., 2007). Although genetic group matched phenotype in most cases, the hybrids often did not display an intermediate morphology and tended to show typical paternal traits (Kremer et al., 2002; Valbuena-Carabaña et al., 2005, 2007; Curtu et al., 2007; Jensen et al., 2009). Therefore, inferences of hybridization in oaks based on morphological characters may lead to wrong conclusions (Stebbins, 1950; Curtu et al., 2007).

Tree growth and iWUE of the three genetic groups at Somosierra partly reflected the *a priori* ranked of species tolerance to drought: the hybrid QuHy and the submediterranean QuPy displayed larger mean growth rates in both subperiods than the temperate QuPe. The latter exhibited the most pronounced decreasing growth trend during the last decades and the largest iWUE for both subperiods, in accordance with the lower stomatal conductance of this species respect to more drought tolerant oak species such as QuPy (Aranda et al., 2004; Fernández, de Uña et al., 2016). It should also be taken into account that QuPe shows earlier budburst and leave unfolding compared to QuPy and, therefore the growing season of QuPe spans longer than that of QuPy (Aranda et al., 1996) and that could also account for the higher iWUE of QuPe respect to QuPy. However, none of these differences could be statistically confirmed: no significant differences were found among the three genetic groups neither in mean BAI nor in mean iWUE for any of the two subperiods under study.

According to our results, QuHy was the only genetic group not displaying a significant declining growth during the recent decades and showed the largest mean growth rates, which could point to more vigor. However, the difference in growth rates respect to the pure species was not statistically significant. Thus, if admixture of two *a priori* distinct gene pools, such as those of a temperate and submediterranean oak species confers any sort of competitive advantage, the difference respect to the pure species was not large enough to be statistically significant in the functional traits analyzed in this study with the exception of the recent growth trend during the most recent decades. Furthermore, we could not even find statistically significant differences neither in BAI nor in iWUE among the two pure species. Previous studies have reported both significant (Ponton et al., 2001, 2002) and non-significant (Thomas and Gausling, 2000; Roussel et al., 2009a; Renninger et al., 2014) differences in iWUE between co-occurring oak species. Differences in iWUE among genotypes and in closely related species such as *Q. robur* and *Q. petraea* seemed to be caused by differences in stomatal conductance (i.e., ratio stomatal density/stomatal area index) rather that in assimilation rate (i.e., traits related to photosynthetic capacity) (Ponton et al., 2002; Roussel et al., 2009b). Stomatal density is not necessarily linked to genotype and displays a high inter-tree and intra-tree variability partly due to environmental conditions such as surface solar radiation, drought and even atmospheric pollution (Ponton et al., 2002; Bruschi et al., 2003; Batos et al., 2010). However, closely related species confronting the same climate constraints or adaptative challenge may also show a similar response or phenotypic adaptation using the same genes (Stern, 2013; Yeaman et al., 2016). Such evolutionary convergence is less likely in highly polygenic traits, however, the partial genetic control of iWUE seem to be oligogenic and explained by a reduced number of quantitative trait loci (Brendel et al., 2008). Considering that the two oak species of Somosierra forest have been coexisting for centuries, the evolution of alleles transferred from one species into another by hybridization process or common phenotypical adaptations in response to more challenging environmental conditions, may explain the similarities in growth rates and iWUE.

### Climate Change-Induced Species Growth Convergence

In line with the non-significance difference in growth rates among genetic groups, the climate–growth relationship was also very similar across species in both subperiods. In contrast, although we could not find significant differences in iWUE among species, the δ^13^C-climate relationship was not always synchronized among groups. δ^13^C was controlled by precipitation during the period 1890–1915 and such influence was species-dependent (a different season in each genetic group). However, during the latter period δ^13^C was more controlled by summer temperature in the three genetic groups. Thus, more restrictive climate conditions during the recent decades (1.3°C higher mean annual temperature and 98 mm lower total annual precipitation respect to the earlier period), may have not only induced a general increase in iWUE as previously described by other authors for oak species (Saurer et al., 2014; Frank et al., 2015; Fernández, de Uña et al., 2016; Martínez-Sancho et al., 2018), but it has also synchronized the response of the different genetic groups to climate. iWUE is defined as the balance between the photosynthetic rate and the stomatal conductance, thus an increase in iWUE as estimated from δ^13^C is linked to an enhancement of the CO_2_ assimilation and/or decrease in ^13^C discrimination as a consequence of reduced stomatal conductance. Higher summer temperature may reduce stomatal conductance to avoid water loss leading to increased iWUE (i.e., Andreu-Hayles et al., 2011; Voltas et al., 2013; Martínez-Sancho et al., 2018). Thus, changes in climate may have triggered a tighter stomatal control across species and hence synchronized the specie’s response to climate and increasing iWUE.

The evolution of the climate–growth relationship also points to an increase in climate-induced limitations on growth. Tree growth of all three genetic groups displayed similar responses to temperature during the first period and to precipitation during the second period, which points out to a synchronized shift in sensitivity to climate during the last 100 years. Shifts in the sensitivity to climate due to more restrictive conditions in the Iberian Peninsula are not unprecedented. Summer temperatures are no longer exerting a positive influence on tree growth, most likely because the increase in temperature during the last century is becoming stressing as observed in different tree species growing in the Mediterranean (e.g., Andreu et al., 2007; Gea-Izquierdo et al., 2009; Dorado-Liñán et al., 2019). In contrast, precipitation did not have a significant influence in none of the species during the earlier period. However, previous year spring precipitation became the main driver for tree growth in all three genetic groups during the second period. Prevailing precipitation effects on oak growth in moisture-limited sites have been consistently described at the western Mediterranean although the most commonly reported relates to current year late spring-summer precipitation (Mérian et al., 2011; Michelot et al., 2012; Gea-Izquierdo and Cañellas, 2014; Dorado-Liñán et al., 2017; Martínez-Sancho et al., 2018) and previous year spring is usually not considered in the analyses. Precipitation in previous year spring was detrimental for tree growth in Somosierra, most likely because reduced solar radiation and low temperature prevents budbreak and leaf unfolding (Dantec et al., 2014), hence reducing the length of the growing season and the amount of photosynthates that can be stored (Fritts, 1976). This points to the existence of interannual carry-over effects in the climatic legacy (Anderegg et al., 2013, 2015). Indeed, growth–climate relationships have also evidenced enhanced lag effects in all three genetic groups during the second period and under warmer and dryer climate. The enhancement of lag effects could be a beneficial adaptive measure in stressed environment at expenses of a growth reduction as a trade-off (McDowell et al., 2008). Such tree growth-climate lag response has been widely described in climate-limited forests (e.g., Barber et al., 2000; Girardin et al., 2016; Martínez-Sancho et al., 2018).

### The Sign of the Growth-iWUE Relationship

The growth convergence among the genetic groups due to changes in climate was further confirmed by the results of the GLMM. A single GLMM developed for all three genetic groups displayed a larger percentage of explained variance and better predicting skills for the second period than for the first one, denoting that the common variability among groups increased during the period 1980–2015 respect to 1880–1915. The model also confirmed the change on climate drivers of tree growth observed in the correlation with climate. However, it also evidences the change on the influence on iWUE on growth.

During the period 1880–1915, iWUE was positively influencing tree growth. However, the conspicuous positive increase of iWUE over the second study period 1980–2015 did not stimulate tree growth as revealed by the GLMM. Indeed, during the second period, the inclusion of iWUE as a predictor did not improve the GLMM model containing only climatic predictors. This result is not unprecedented since increase in iWUE has been associated to sustained or even reduced growth in conifer and broadleaf species in many tree-ring based studies (e.g., Andreu-Hayles et al., 2011; Maseyk et al., 2011; Bader et al., 2013; Voltas et al., 2013; Granda et al., 2014; Fernández, de Uña et al., 2016; Martínez-Sancho et al., 2018) suggesting a prevailing reduction in stomatal conductance rather than an enhancement of carbon assimilation photosynthetic rates in response to increasing atmospheric CO_2_ concentration (Maseyk et al., 2011; Voltas et al., 2013). Our results also evidenced that the sign of the growth–iWUE relationships is individual-tree dependent (i.e., model accounting for the variation in individual tree response to the slope of iWUE explained higher variance than those accounting for genotype or phenotype). Unlike the first period when the influence of iWUE on growth was always positive, both positive and negative coefficients were observed for the influence of iWUE on BAI during the second period.

Intraspecific variability in interannual growth and iWUE and hence, in the sign of the growth–iWUE relationship, has two main sources of variability: environment and genotype. Individual tree response to drought have been described to be closely linked to the tree water balance (i.e., mass balance of atmospheric demand versus moisture reservoir; Berdanier and Clark, 2018) and that sunlight can be the main limiting factor for ^13^C discrimination (Ponton et al., 2002; Rodríguez-Calcerrada et al., 2008). Thus, access to moisture reservoir to maintain transpiration under drought as well as leaf position and the ability to acclimate the photosynthetic system under changing light intensity conditions might be determinant for the sign of the BAI–iWUE relationship.

Our results also showed that intraspecific variability in iWUE was close in range to the interspecific variability. Despite our reduced sample size, this result is not unprecedented: isotopic discrimination among genotypes of *Q. robur* have been described to represent 3‰ (40% in iWUE) in common garden experiments (Brendel et al., 2008; Roussel et al., 2009a, b), which exceeds the 1‰ interspecies *Q. petraea* and *Q. robur* variability described for both mixed forest and garden experiments (10% in iWUE; Ponton et al., 2001, 2002). However, to draw conclusion on this regard, isotopic analyses should be performed in a larger number of trees than the one used for the purpose of this study.

## Conclusion

Tree growth of the three defined genetic groups followed the *a priori* rank of specie’s tolerance to drought, being the temperate QuPe the species displaying a lower growth rate than the submediterranean QuPy and the hybrid. However, that was not enough to yield significant differences in growth rates among the three genetic groups neither during the early period 1880–1915 (and theoretically more favorable climate conditions for QuPe) nor during the late period 1980–2015 (theoretically more suitable climate conditions for QuPy). Furthermore, tree growth seemed to be driven by the same climatic factors for the three genetic groups in both subperiods. The only difference in the response to climate among groups was observed for the δ^13^C-climate relationship during the earlier period, but not during the late period. During the second period, no greater response to moisture availability was observed in the theoretically less-drought tolerant species QuPe, although it displayed the highest iWUE in accordance with a lower level of conservatism in its hydraulic strategy. However, such a difference was not statistically significant respect to QuPy and QuHy. Thus, climate change seemed to have triggered a tighter stomatal control across genetic groups, increasing the iWUE and coherence of growth during the most recent decades. Yet, the absence of statistical differences in iWUE among genetic groups could also be related to the low sample size used in dendrochemical studies such as this one. Most of the studies aiming at disentangling the influence of genotype-environment interaction shaping trees’ response to climate have been carried out using seedlings, which are known to physiologically differ from adult trees. Mixed oak forests such as the one in our study are invaluable resources to tackle this question while avoiding the “juvenile effect” and even also ontogeny-related issues in old-growth life stage by using a size or age-stratified sampling.

## Data Availability Statement

The datasets generated for this study are available on request to the corresponding author.

## Author Contributions

ID-L and GG-I conceived the study. ID-L, GG-I, and MV-C performed fieldwork and laboratory analyses. ID-L performed the statistical analyses and wrote the first draft of the manuscript. GG-I, MV-C, IC, and LG made comments and suggestion to the manuscript.

## Conflict of Interest

The authors declare that the research was conducted in the absence of any commercial or financial relationships that could be construed as a potential conflict of interest.

## Footnotes

1 http://cdiac.ornl.gov/
