# Supplementary material for: Climate Change Synchronizes Growth and iWUE Across Species in a Temperate-Submediterranean Mixed Oak Forest
Source: Front Plant Sci. 2020 Jun 11;11:706. doi: 10.3389/fpls.2020.00706 (PMC7300280; doi:10.3389/fpls.2020.00706)
Supplement: Supplementary file 1 [file Data_Sheet_1.docx]

**SUPPLEMENTARY MATERIAL**

**Tables**

**Supplementary Table 1**. Predictors included in the GLMM. Superscript numbers 1, 2 indicate random effect included in the GLMM for the first and second subperiod, respectively. Abbreviations: iWUE: intrinsic water use efficiency; DBH: diameter at breast heigh; h: height; SuT: summer temperature; SuP: summer precipitation; SpT: spring temperature; SpP: spring precipitation; WiT: winter temperature; WiP: winter precipitation; AuT: autumn temperature; AuP: autumn precipitation; SuTt/SuPt: previous year summer temperature/precipitation; SpTt/SpPt: previous year spring temperature/precipitation; WiTt/WiPt: previous year winter temperature/precipitation; AuTt/AuPt: previous year autumn temperature/precipitation.

**Supplementary Table 2*.*** Correlation among iWUE series for the two subperiods. Values in brackets correspond to the value of correlation after removing the trend using a linear regression model. Bold font denotes significant correlation at 99% level.

**Supplementary Table 3**. Coefficients from the GLMM for the two periods under study. Abbreviations: Sp:genetic group; iWUE: intrinsic water use efficiency; SuP: summer precipitation; SpP: spring precipitation; SuTt: previous year summer temperature; WiT: winter temperature; DBH: diameter at breast heigh; SuPt: previous year summer precipitation; SpT: spring temperature; WiP: winter precipitation; WiT: winter temperature; WiPt: previous year winter precipitation.

**Supplementary Table 4**. ANOVA results comparing the final GLMM for the period 1880-1915 with different random effects. Df: number of parameters; AIC: Akaike information criterion; BIC: Bayesian information criterion; Chisq: Chi-square test; Pr(>Chisq): Chi-square test p-value. Abbreviations as in STable 3. Bold font indicates selected model based on the lowest AIC.

**Supplementary Table 5**. ANOVA results comparing the final GLMM for the period 1980-2015 with different random effects. Abbreviations as in STable 3,4. Bold font indicates selected model based on the lowest AIC.

**Figures**

*
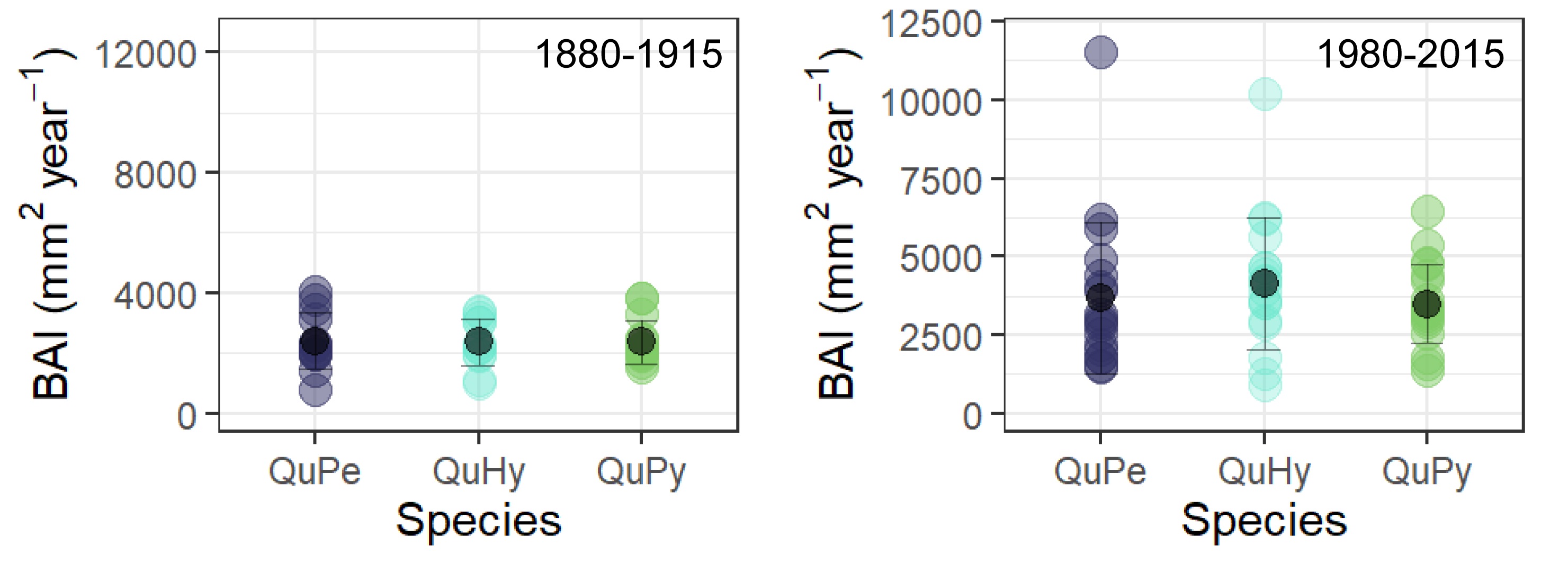
*

**Supplementary Figure 1**. Differences in BAI between genetic groups for the two subperiods under study. QuPe: *Quercus petraea*; QuHy: *quercus* hybrids; QuPy: *Q. pyreanica*. No significant differences were found.


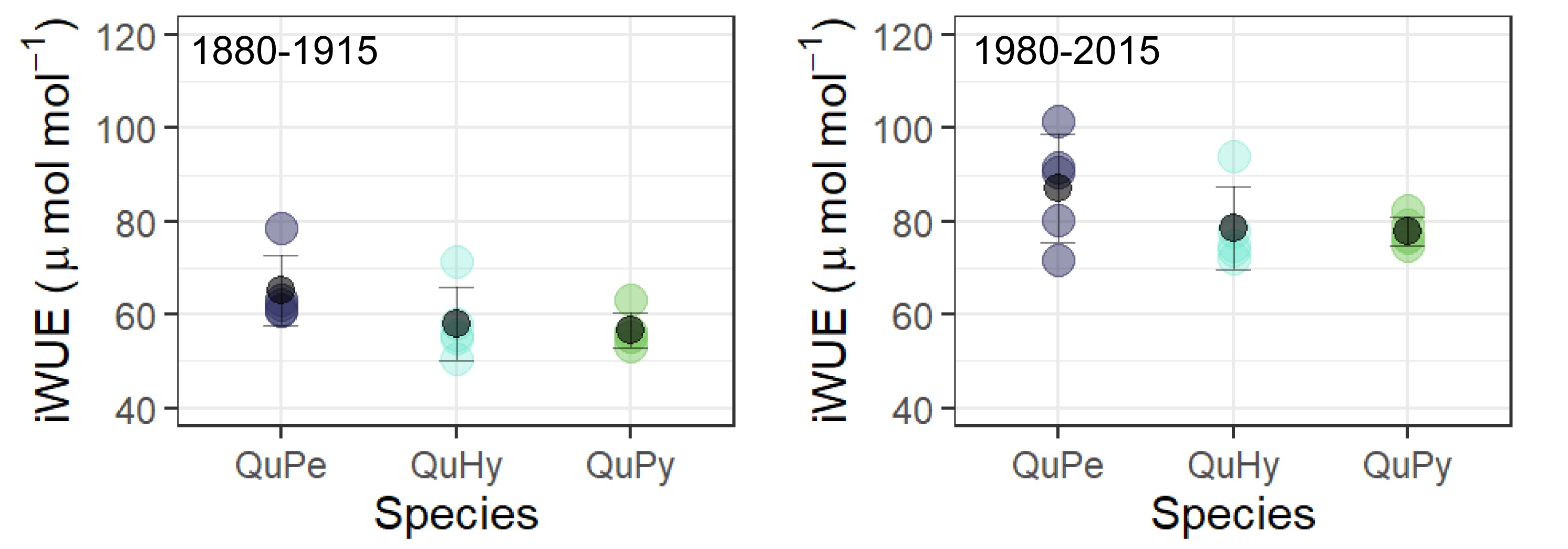


**Supplementary Figure 2**. Differences in iWUE among genetic groups for the two subperiods under study. QuPe: *Quercus petraea*; QuHy: *quercus* hybrids; QuPy: *Q. pyreanica*. No significant differences were found.

*
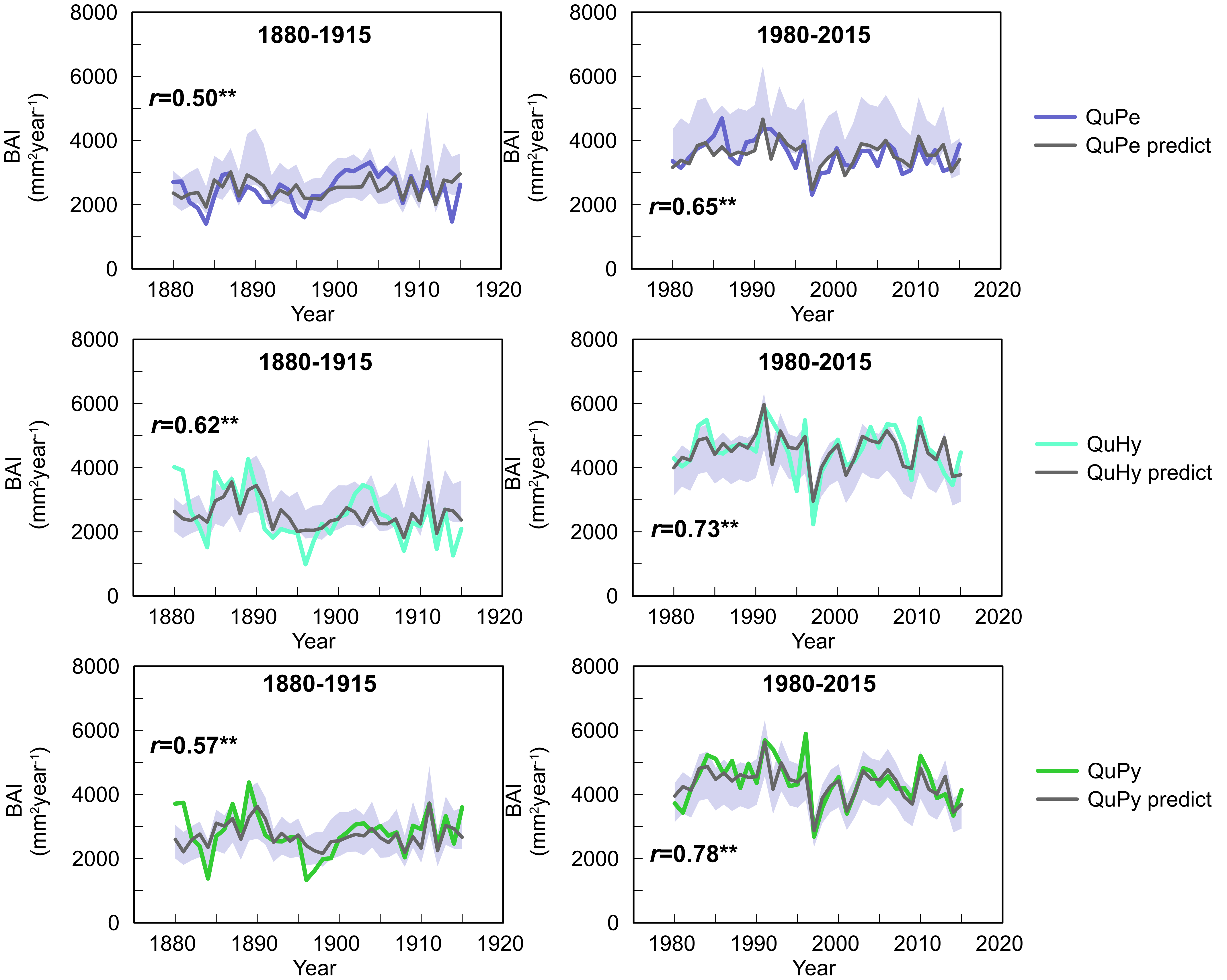
*

**Supplementary Figure 3**. Observed (coloured line) and predicted (grey line) basal area increment for each genetic group based on the GLMM developed for each period. QuPe: *Quercus petraea*; QuHy: *quercus* hybrids; QuPy: *Q. pyrenaica*. Grey area indicates the 95% confidence intervals of prediction. Value of correlation between observed and predicted BAI series is shown. **denotes significance at 99% level
